# Unveiling Plant Metabolic Diversity: Integrating Metabolomics with Multi-Omics Approaches for Crop Improvement

**DOI:** 10.3390/plants15050846

**Published:** 2026-03-09

**Authors:** Xin Song, Bing-Liang Fan, Xixiong Hong, Peipei Su, Minshan Sun

**Affiliations:** 1 School of Life Science and Environmental Resources, Yichun University, Yichun 336000, China; 207209@jxycu.edu.cn (X.H.); ppsu886@163.com (P.S.); 2 State Key Laboratory for Conservation and Utilization of Subtropical Agro-Bioresources, College of Life Science and Technology, Guangxi University, Nanning 530004, China; blfan@st.gxu.edu.cn; 3 College of Plant Protection, Henan Agricultural University, Zhengzhou 450002, China

**Keywords:** metabolomics, multi-omics, crop improvement, metabolite regulation, integrative analysis

## Abstract

Metabolomics, representing the biochemical phenotype of cells or tissues, serves as an intrinsic factor underlying the differences in plant traits. Recent advances in multi-omics technologies have significantly deepened our understanding of plant metabolic diversity, enabling researchers to dissect complex biochemical networks at unprecedented levels of detail. This review explores the integration of metabolomics with genomics, transcriptomics, proteomics, epigenomics, microbiomics, and other omics approaches, emphasizing the power of these combined approaches in unraveling the molecular mechanisms underlying plant adaptation, stress resistance, and phenotypic variation. Through a critical analysis of representative case studies across diverse crops, we demonstrate how multi-omics strategies facilitate the identification of key metabolic pathways and regulatory networks for crop improvement. We also discuss current challenges in data integration, metabolite coverage, and the functional characterization of unknown compounds, and propose future directions for overcoming these limitations. Addressing these challenges will require both the enhanced resolution and sensitivity of analytical techniques, as well as more robust frameworks for data integration and interpretation. By overcoming these challenges, the convergence of metabolomics with other omics disciplines will continue to expand our understanding of plant biology, offering novel insights and innovation in crop breeding and sustainable agriculture.

## 1. Introduction

Plant metabolism is notably diverse, with estimates suggesting that the plant kingdom contains between 200,000 and 1 million metabolites, the majority of which are classified as secondary (or specialized) metabolites [1,2]. Primary metabolites, such as amino acids, sugars, and nucleic acids, are essential for growth and are universally produced across plant species. In contrast, secondary metabolites—while not required for basic cellular function—play critical roles in plant interactions with the environment. These compounds, which have been harnessed by humans for thousands of years for purposes ranging from fragrances and flavorings to pigments, insecticides, and medicinal agents [3], contribute significantly to a plant’s ability to adapt to environmental conditions, such as natural disasters, pest attacks, and herbivory.

The diversity of metabolites is thought to represent a key evolutionary strategy by which plants adapt to various ecological niches. These metabolites are key in biotic and abiotic stress resistance, enabling plants to defend against threats while also aiding reproduction and dispersal [4,5]. For instance, individual plant species may produce upwards of 5000 different metabolites, reflecting the remarkable diversity and adaptability of plant metabolism [6]. This metabolic diversity evolves continually, enhancing plant survival under adverse conditions. Secondary metabolites, including terpenoids, phenylpropanoids, and alkaloids, not only serve essential functions in stress resistance but also hold significant pharmacological and industrial applications [7]. While the core metabolic pathways in plants are conserved across many species, similar to those in non-plant organisms, plants and fungi possess an additional capacity to synthesize a vast repertoire of specialized compounds, which make up the bulk of their metabolomes. This unique metabolic ability offers a compelling framework for studying the evolutionary processes that contribute to biological complexity. Recent advances in molecular genetics, biochemistry, and structural biology have shed light on the biosynthesis of specialized metabolites in plants [8], revealing how the evolution of metabolic networks contributes to plant adaptability and survival in diverse environments, thereby reinforcing the notion that metabolite diversity is a key strategy for thriving in complex ecosystems.

The development of comprehensive analytical techniques, including genomics, transcriptomics, metabolomics, and other omics, have significantly advanced our understanding of plant metabolic diversity. These approaches have unveiled the complex molecular mechanisms plants use to regulate their chemical composition, revealing that the variation in metabolism within a single species is far greater, both qualitatively and quantitatively, than previously assumed [9]. In particular, genomics, epigenomics, and metabolomics, have facilitated the identification of genes responsible for metabolite modifications, shedding light on the molecular basis of secondary metabolism [8]. Among these, metabolome genome-wide association studies (mGWAS) significantly improve the identification of metabolite-regulating genes and elucidate the metabolic pathways underlying plant phenotypic traits. For instance, mGWAS have uncovered the genetic architecture and key genes underlying the diversity of oil composition in rice grains [10], while natural polymorphisms in the transcription factor *ZmICE1* have been identified through association studies as critical regulators of amino acid metabolism, contributing to cold tolerance in maize [11]. Furthermore, metabolite-based epigenetic-wide association study (mEWAS) extends this analysis to epigenetic modifications, such as DNA methylation, providing a more comprehensive understanding of metabolic regulation. However, despite these advances, challenges remain, including the complexity of plant genomes, genetic redundancy, and the intricate regulatory networks that govern secondary metabolism [12,13,14,15].

This review provides a comprehensive overview of integrating metabolomics with genomics, transcriptomics, epigenomics and other omics in plant science. We focus on the integrative logic that links multi-omics data to metabolic pathways for precision crop improvement. By examining the synergistic effects of these integrated approaches, we aim to elucidate the multi-layered regulatory mechanisms—from genetic variation to dynamic molecular responses—that underlie plant metabolite diversity and, ultimately, phenotypic traits. Furthermore, we will discuss the current challenges and future prospects of multi-omics research in plant science, focusing on how these approaches can enhance the understanding of plant biology and contribute to agriculture and pharmaceuticals.

## 2. Recent Advances in Metabolomics Research

### 2.1. Development of Metabolomics

To map the field’s progression, we analyzed literature trends using keyword searches (‘plant metabolomics’, ‘multi-omics’, ‘crop’) in major databases including Web of Science and PubMed, prioritizing impactful studies from the past decade that demonstrate integration. Inclusion criteria were: (1) peer-reviewed original research or reviews published between 2014 and 2024; (2) studies integrating metabolomics with at least one other omics approach; (3) research on crop species or model plants; (4) articles with a clear methodology and data availability.

**Figure 1:**
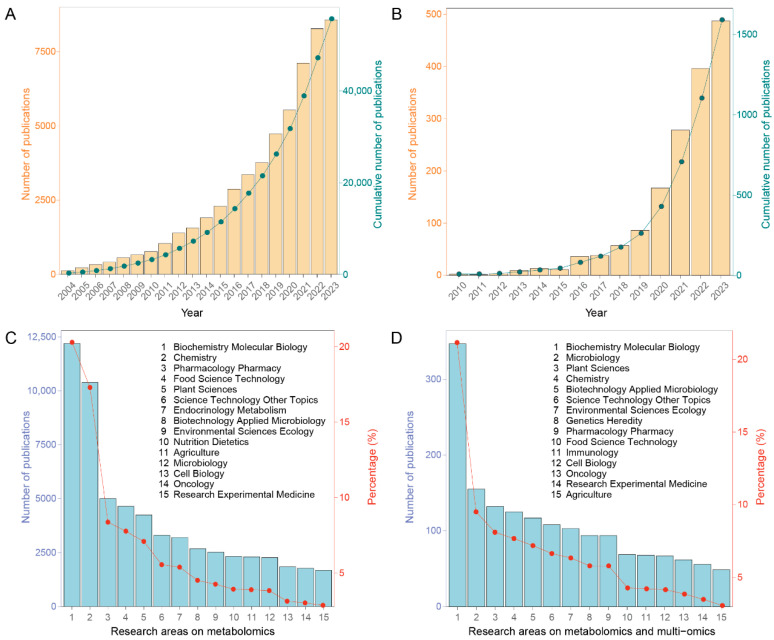
Literature search on metabolomics and multi-omics from 2004 to 2023. Number of publications per year and cumulative number of publications on (**A**) metabolomics and (**B**) metabolomics and multi-omics. The integration of metabolomics with multi-omics first appeared in publications in 2010, with no prior data available. Number of publications of the top 15 research areas on (**C**) metabolomics and (**D**) metabolomics and multi-omics.

Over the past 20 years, metabolomics has emerged as a rapidly expanding field, with more than 50,000 articles published (Figure 1). From 2010 to 2023, the number of publications on metabolomics and its integration with other omics has steadily increased, growing from just a few publications in 2010 to nearly 500 in 2023 (Figure 1A,B). This trend highlights the rising interest in metabolomics and multi-omics research. The top 15 research areas in metabolomics are predominantly concentrated in biochemistry molecular biology (20.21%), chemistry (17.22%), and pharmacology pharmacy (8.28%) (Figure 1C). A similar distribution is observed when considering metabolomics and multi-omics together, with biochemistry molecular biology comprising the largest share at 21.08%, while other research areas each account for less than 10% (Figure 1D). Notably, the combination of metabolomics with multi-omics began to emerge in 2010, with a notable increase in articles related to microbiology, where the proportion of papers jumped from 3.76% to 9.42% after incorporating the keyword ‘multi-omics’, indicating a strong association between metabolomics and microbiology. This shift underscores the evolving focus from purely analytical chemistry towards biological and ecological applications of metabolomics.

### 2.2. Classification of Metabolomics Approaches

In plant research, metabolite analysis methods can be categorized into two main approaches: targeted and untargeted. Targeted metabolomics focuses on quantifying specific metabolites by comparing them to known standards, offering high sensitivity and specificity. This approach is particularly suitable for hypothesis-driven studies, especially when the target metabolites are well defined. In contrast, untargeted metabolomics seeks to detect and identify as many metabolites as possible without prior knowledge of their identities, providing a holistic view of the metabolome and facilitating the discovery of novel metabolites and metabolic pathways. Various analytical techniques are employed in metabolomics (Table 1), such as gas chromatography–mass spectrometry (GC-MS) [16], liquid chromatography–mass spectrometry (LC-MS) [17], nuclear magnetic resonance (NMR) [18], and direct infusion mass spectrometry (DIMS) [19]. While GC-MS and LC-MS are widely used for their sensitivity and versatility, NMR offers non-destructive analysis of samples and DIMS enables rapid metabolite profiling. Building upon these foundational techniques, many other metabolomics methods have been developed to enhance analytical capabilities (Table 1). Despite their strengths, no single technique can provide comprehensive coverage of the metabolome, necessitating the integration of multiple complementary technologies.

### 2.3. Applications and Challenges of Metabolomics

Metabolomics has significantly contributed to biological insights, ranging from biomarker discovery to systems biology, elucidating numerous biochemical pathways and demonstrating that metabolism is genetically guided by genes similar to those influencing other heritable traits [28,29,30]. In plants, metabolomics-based approaches have been used to predict crop yield and quality traits, understand the mechanisms of heterosis, and identify bioactive compounds in plant extracts [31,32]. The ability to analyze plant metabolites at such a detailed level has led to new discoveries in plant biology and enhanced agricultural research. Despite progress, the field of metabolomics continues to face challenges, primarily due to the complexity of metabolite structures and the broad concentration ranges within biological samples [27]. Comprehensive metabolic analysis using a single platform remains unattainable, as each technology has its limitations in sensitivity, specificity, or metabolite coverage. To overcome this, the integration of multiple complementary (orthogonal) technologies or the incorporation of multi-omics approaches can better elucidate biological traits. Notably, differences in experimental design (e.g., growth conditions, sampling time) across studies can affect metabolic profiles and complicate direct comparisons. This integrated analytical strategy is crucial for fully harnessing the potential of metabolomics in biological and agricultural research.

## 3. Combining Methods with Multi-Omics to Analyze Metabolic Regulation

### 3.1. Natural Variation Leads to Metabolic Diversity Within Plant Populations

Genomics plays a pivotal role in advancing our understanding of the structural and functional dynamics of plant genomes. By identifying key genes and genetic variants, particularly structural variations and SNPs, genomics provides essential molecular targets for crop trait improvement. Metabolites, in turn, are intricately interconnected due to the co-regulation of biosynthetic pathways, shared reactions within metabolic networks, common modifications of structurally similar compounds, and competition for substrates in various enzymatic processes [33,34]. This interconnectedness underscores the complexity of metabolic regulation and highlights the importance of integrating genomics with metabolomics for a comprehensive understanding of plant biology. To illustrate the integration of genomics and metabolomics in crop improvement, several studies serve as prime examples of how mGWAS and metabolome-based quantitative trait locus (mQTL) analyses have been instrumental in directly linking genetic variation to biochemical phenotypes, thereby uncovering the metabolic basis of complex traits, including adaptive responses to environmental stresses.

**Rice**: mGWAS of seed glycerolipids across diverse accessions identified *OsLP1* as a key gene controlling saturated triacylglycerol levels [35]. Notably, allelic variation in *OsLP1* was co-selected with a flooding tolerance QTL, linking lipid metabolism to submergence adaptation.

**Wheat**: Comprehensive mGWAS in wheat kernels dissected the genetic architecture of flavonoid decoration pathways, identifying 387 metabolites associated with over 14,600 polymorphic SNPs and 26 candidate genes related to agronomic traits [36]. Functional validation confirmed glycosyltransferases and malonyltransferases as key regulators of antioxidant flavonoid modification.

**Maize**: Integrated genomics and metabolomics identified genes and biomarkers associated with salt–alkali tolerance [37], revealing the role of compatible solutes and flavonoids in osmotic adjustment.

**Additional crops**: Similar approaches have elucidated drought resistance mechanisms in tomato [38], flavonoid synthesis in citrus [39], fruit quality in apples [40] and high α-linolenic acid content of seed oil in perilla [41]. While these associations are powerful, their validation often requires functional studies to confirm causality.

These examples demonstrate how integrating genomics and metabolomics through mGWAS and mQTL analyses has significantly advanced our understanding of the genetic and biochemical mechanisms regulating complex traits in crops. Beyond providing insights into metabolic regulation, these studies hold practical value for crop improvement, enabling more targeted and efficient breeding strategies to enhance productivity, quality, and resilience. Together, these studies demonstrate how linking the genome to the metabolome provides a direct, causal entry point into the genetic architecture of metabolic regulation, revealing key genes that serve as levers for trait manipulation.

### 3.2. Comparative Transcriptome Analyses of the Plant Metabolic Kingdom

Transcriptomics can be used to analyze the full spectrum of RNA molecules expressed in cells, providing insights into gene expression and regulatory networks [42]. While transcriptomics offers insights into gene expression dynamics, it is limited in directly linking these changes to phenotypic outcomes. Metabolomics, in contrast, captures the full spectrum of small-molecule metabolites within a sample, establishing a direct connection to the phenotype and often amplifying minor gene expression changes into significant metabolic shifts [43]. The integration of transcriptomics and metabolomics provides a robust framework for investigating the complex regulatory networks governing biological processes, which is especially valuable for studying plant growth and development, where transcription factors play crucial regulatory roles. In multi-omics association analysis, the primary focus is on correlating differentially expressed genes (DEGs) from transcriptomics with differential accumulated metabolites (DAMs) identified through metabolomics. Techniques such as orthogonal projections to latent structures (O2PLS) and cross-omics association analysis reveal correlations between DEGs and DAMs, visualizing these relationships with heatmaps and network diagrams to highlight significant findings [44].

This integrated approach is powerfully illustrated in studies of stress responses. In maize, combined multi-omics analysis of autophagy-deficient mutants under fixed-carbon starvation revealed that the absence of autophagy led to stark metabolic reprogramming, characterized by the accumulation of free amino acids and nitrogen-rich nucleotide catabolites, alongside the upregulation of corresponding catabolic pathways [45]. This demonstrates how autophagy is essential for nitrogen remobilization and homeostasis during severe energy stress, preventing the wasteful accumulation of metabolic intermediates and adjusting respiratory substrate choice. Similarly, a transcriptomic and metabolomic study comparing freezing-tolerant and sensitive potato species identified salicylic acid (SA) as a key regulator [46]. The study showed that cold-induced SA accumulation in the tolerant species activated downstream signaling (e.g., via *HSFC1*), which in turn coordinated the upregulation of genes in protective pathways, leading to the synthesis of specific stress-responsive metabolites. This provides a direct mechanistic link between a hormone signal, transcriptional reprogramming, and the metabolic basis of enhanced constitutive freezing tolerance.

The power of integration equally illuminates the molecular basis of developmental quality traits. Research on passion fruit ripening combined genomics, transcriptomics, and metabolomics to pinpoint that the substantial accumulation of flavor- and color-determining metabolites, such as specific terpenes and anthocyanins, was governed by the activity of differentially expressed biosynthetic gene clusters [47]. The functional characterization of terpene synthase genes directly linked sequence variation to distinct aromatic profiles. This approach decodes how the transcriptional regulation of metabolism directly shapes critical consumer-oriented fruit attributes. Furthermore, studies on cold stress in rapeseed [48] and peanut [49] have consistently shown that cold resilience is associated with reprogrammed amino acid and flavonoid metabolism, linking specific transcriptional cascades to the production of osmoprotectants and antioxidants. Altogether, this integrative approach not only enhances our understanding of plant biology but also supports the development of crop varieties adapted to various environmental conditions and agricultural needs. Thus, this integration deciphers the dynamic, information-processing layer of regulation, connecting transcriptional changes to their functional metabolic outcomes and identifying key regulatory hubs (e.g., transcription factors) within biological processes.

### 3.3. EWAS Emerge as a Novel Approach to Elucidate Plant Metabolism

Epigenome-wide association studies (EWAS) are a powerful approach used to identify associations between epigenetic modifications, such as DNA methylation, and phenotypic traits or diseases [50]. Unlike traditional genome-wide association studies (GWAS), which focus on genetic variants, EWAS investigate how epigenetic changes, often influenced by environmental factors, can regulate gene expression without altering the underlying DNA sequence. One of the key characteristics of EWAS is their ability to capture dynamic epigenetic modifications that may vary over time, tissues, or environmental conditions. This provides an added layer of complexity to understanding phenotypic traits that cannot be explained solely by genetic variants. Unlike the relatively static nature of germline mutations targeted by GWAS, epigenetic marks are reversible and modifiable, which allows for the possibility of more adaptive responses to external stimuli. The limitations of traditional GWAS are becoming increasingly evident, particularly in their inability to fully explain the missing heritability of complex traits. While GWAS has identified numerous genetic loci associated with various diseases, these loci often account for only a small fraction of the expected genetic contribution to the trait [51]. EWAS hold several advantages in the context of metabolomics and the study of metabolic traits. Since metabolism is highly influenced by environmental factors, epigenetic regulation plays a critical role in metabolic pathways and homeostasis. EWAS can provide insights into how epigenetic changes modulate the expression of genes involved in metabolism, offering a more comprehensive understanding of metabolic phenotypes. By integrating EWAS with metabolomics, researchers can uncover epigenetically driven alterations in metabolic pathways that contribute to disease or adaptability, particularly in complex traits where both genetic and environmental factors play a crucial role [52].

Early proof-of-concept in the model plant *Arabidopsis thaliana* demonstrated the predictive power of epigenomics. Using DNA methylation profiles to predict plant height, a study achieved a predictive correlation of 0.53 and estimated that epigenetic variation accounted for approximately 65% of the phenotypic variance in that population [53]. This established that epigenetic information could substantially contribute to the prediction of complex quantitative traits, independent of genetic sequence. This foundational work paved the way for more comprehensive studies in crops. A landmark multi-omics study in tomato constructed a population-level map integrating the genome (variome), methylome, and metabolome [54]. The analysis revealed that over 80% of differentially methylated regions (DMRs) were not tightly linked to underlying genetic polymorphisms (SNPs), indicating a substantial layer of independent epigenetic regulation. Crucially, by associating both SNPs and DMRs with metabolic profiles, the study showed that methylation variation, particularly hypomethylation at promoters of key biosynthetic genes (e.g., in the flavonoid pathway), could directly explain metabolic diversity and had been shaped during domestication. This demonstrates that epigenetic markers complement genetic markers in deciphering the basis of metabolic traits.

The translational potential of such insights is being actively explored in breeding. For example, incorporating epigenetic markers like DNA methylation into genomic prediction models has been proposed to improve the selection accuracy for complex agronomic traits in soybean, such as yield and seed quality [55]. This strategy leverages epigenetic information to capture the plastic component of phenotypes influenced by environmental history. Despite its potential, EWAS in plant systems face several important limitations. First, epigenetic marks can be unstable across generations and environmental conditions, complicating the interpretation of stable phenotypic associations. Second, distinguishing causal epigenetic modifications from those merely correlated with phenotypic changes remains challenging, particularly without intervention studies. Third, the high cost and technical complexity of whole-genome bisulfite sequencing limit its application in large populations.

In summary, integrating epigenomic and metabolomic data, alongside other omics, holds considerable potential for advancing our understanding of complex traits in plant diseases. This approach uncovers an environmental-adaptive layer of regulation, demonstrating how epigenetic modifications can modulate metabolic pathways independently of the DNA sequence, offering another dimension for understanding phenotypic plasticity.

### 3.4. Protein–Metabolite Interactions Modulate Plant Phenotypes

The protein–metabolite nexus encompasses three fundamental mechanisms: direct enzyme–substrate interactions driving metabolic flux, allosteric regulation of protein activity by metabolites, and protein-mediated metabolite transport and compartmentalization. Understanding these interactions is essential for deciphering how proteomic changes translate into metabolic phenotypes.

Proteomics explores the complete protein complement of a genome, focusing on protein expression, interactions, modifications, and functions. Advanced techniques, including mass spectrometry [56], quantitative labeling methods [57], and structural analyses [58], provide detailed insights into protein dynamics and their roles in plant traits and responses to environmental factors. The binding affinity of various small molecules to proteins can significantly influence metabolic pathways and regulatory networks, thereby affecting overall metabolomic profiles. This interplay between small molecules and proteins highlights the critical relationship between proteomics and metabolomics, emphasizing how variations in protein function and interactions can shape metabolic responses and adaptations in plants [59]. The joint analysis of proteomics and metabolomics provides a comprehensive approach to understanding cellular activities, revealing molecular regulatory mechanisms that drive phenotypic changes. This integration facilitates cross-validation and identifies key biomolecules and pathways, effectively reducing background noise in genetic information transmission.

This integrated approach has been instrumental in elucidating complex physiological processes. For instance, a systems biology study on lignin biosynthesis in *Brachypodium distachyon* combined proteomics and metabolomics to reveal that abundant ammonia-lyase proteins not only drive lignin synthesis but also mediate a critical metabolic link: the released ammonia is recycled into amino acid synthesis, particularly glutamine [60]. This discovery illustrates how a key biosynthetic protein family coordinates carbon allocation for structural polymer formation with nitrogen availability for primary growth, demonstrating a fundamental protein–metabolite nexus at the heart of plant resource partitioning.

Similarly, integrated proteomics and metabolomics have precisely mapped molecular toxicity pathways under chemical stress. In wheat seedlings exposed to the antibiotic, florfenicol, the combined analysis revealed a severe downregulation of photosynthetic proteins, corresponding to disrupted chloroplast ultrastructure, alongside a significant upregulation of proteins involved in the tricarboxylic acid (TCA) cycle and oxidative stress response [61]. Concomitantly, metabolomics showed the accumulation of TCA cycle intermediates and amino acids. This coordinated response indicates a wholesale metabolic shift from photosynthesis to respiration and cellular detoxification under chemical insult.

The power of this strategy is equally evident in deciphering environmental adaptation. A study on apple fruit ripening at different altitudes revealed that high-altitude conditions induced the accumulation of anthocyanins and other phenolic compounds in the peel, which was associated with elevated levels of specific carbohydrates and distinct changes in the abundance of photosynthetic and stress-related proteins [62]. Furthermore, this approach is key to diagnosing developmental phenotypes. An investigation into the yellow leaf mutant of tea plant combined metabolomics and proteomics to show that the phenotype resulted from a coordinated inhibition of chlorophyll biosynthesis proteins and a deficiency in photosynthetic proteins, coupled with an upregulation of alternative pathways like carotenoid and phenylpropanoid biosynthesis [63]. Additionally, a study on *Arabidopsis* ecotypes exposed to salt stress revealed significant alterations in key metabolic pathways [64].

Overall, the combined analysis of proteomics and metabolomics enables a more systematic and detailed exploration of biological processes, offering critical insights into gene function, phenotypic effects, and potential applications in molecular mechanism modeling and crop improvement.

### 3.5. Plant Microbiome–Metabolome Interactions in Shaping Metabolic Pathways

Plant microbiomics has unveiled the critical influence of microbial communities on plant health, resilience, and productivity, particularly through root-associated interactions [65]. Metagenomics has enabled the detailed exploration of their composition, function, and interactions under various environmental conditions [66]. The integration of metabolomics and microbiomics provides a comprehensive approach to understanding plant–microbe–environment interactions, particularly under abiotic stress conditions such as drought, heat, nutrient deficiency, and toxic metals in the soil [67].

This integrated approach has elucidated specific mechanisms by which microbes modulate plant metabolism to confer stress tolerance. A seminal study on wild soybean under salt stress demonstrated that salt-tolerant genotypes enriched specific *Pseudomonas* species in their root microbiome [68]. Metabolomic analysis revealed that this beneficial association was driven by the root exudation of purine metabolites, particularly xanthine, which acted as chemoattractants to enhance microbial motility and colonization. This microbe-mediated metabolic pathway directly contributed to the plant’s enhanced salinity tolerance, showcasing a precise molecular dialog between host metabolism and microbiome assembly under stress.

Beyond supporting plant physiology directly, certain *Pseudomonas* species contribute to environmental remediation by degrading hydrocarbon pollutants [69,70]. Their capability and direct relevance to plant metabolic engineering lies in their shared metabolic pathways [71]. Deciphering such networks aligns with the integrative multi-omics approaches central to this review, highlighting how understanding microbial metabolism extends from plant stress tolerance to ecosystem recovery.

Similarly, research on legume–rhizobia symbiosis under varying nitrogen conditions has highlighted how microbial signals reshape root metabolism [72]. The production of bacterial Nod factors not only initiated nodulation but also systemically reprogrammed root exudate composition, which in turn selectively shaped the broader rhizosphere microbial community. This illustrates a feedback loop where microbial activity alters the plant’s metabolic landscape, which then further modulates the microbiome for nutrient acquisition. The translational potential of harnessing this interplay is evident in studies using beneficial metabolites to recruit stress-alleviating microbes. For example, in apple trees under cadmium stress, the exogenous application of dopamine altered root metabolite profiles, selectively enriching for beneficial microbial taxa [73]. This recruited microbiome-enhanced host antioxidant capacity and photosynthesis, effectively mitigating cadmium toxicity. Parallel findings in poplar showed that natural variation in root metabolite profiles could modulate the microbiome to promote growth, suggesting an inherent metabolic strategy for microbiome management [74]. Furthermore, this microbiome–metabolome axis is crucial in plant–pathogen interactions. In tomatoes, resistant and susceptible cultivars were found to exude distinct sets of root metabolites upon pathogen challenge, which directly inhibited pathogen growth or enriched beneficial fungi, respectively [75]. This indicates that disease resistance traits are in part mediated by the plant’s ability to steer its microbiome through specific metabolic outputs.

Overall, the integration of metabolomics and microbiomics offers a multi-dimensional understanding of plant growth mechanisms and potential strategies for optimizing cultivation practices and rehabilitating stressed agro-environments. Therefore, integrating microbiomics expands the regulatory network beyond the plant genome, revealing how host metabolism and microbial community assembly are interlinked to form an extended phenotype crucial for health and adaptation.

### 3.6. Integrating Metabolomics with Other Omics Fields

Ionomics explores the distribution and regulation of elemental composition in organisms and offers valuable insights into how elements function in cellular physiology and ecological adaptation [76]. Integrating ionomics with metabolomics enables a deeper understanding of how elemental profiles influence metabolic processes. For example, studies on microgreens have revealed distinct metabolite and mineral profiles compared to mature plants, with higher levels of bioactive compounds such as phenolics and essential minerals like selenium and zinc, highlighting their enhanced nutritional value [77]. Additionally, research on platinum nanoparticles (PtNPs) in rice demonstrated their accumulation in plant tissues, causing oxidative stress and iron homeostasis disruption, illustrating the environmental significance of ionomics [78]. Furthermore, ionomics has uncovered plant–microbe interactions, such as the role of lactic acid bacteria in improving potassium uptake and reducing aluminum toxicity in cassava [79]. In agricultural systems, ionomics has been used to study the impact of cultivation practices, with multi-omics approaches revealing the contribution of organic nitrogen to crop yield formation, emphasizing the value of integrating ionomics with other omics to promote sustainable agriculture [80].

Spatiotemporal metabolomics represents a powerful approach to understanding the dynamic distribution of metabolites within plants across different tissues and developmental stages. Traditional metabolomics methods, such as GC-MS and LC-MS, often disrupt the spatial context of metabolites during sample preparation, making it challenging to discern the underlying regulatory mechanisms within plant components. In contrast, spatial metabolomics, through techniques like matrix-assisted laser desorption ionization–mass spectrometry imaging (MALDI-MSI), directly measures the spatial distribution of metabolites, providing a more accurate representation of their localization within plant tissues [81]. This approach also facilitates the exploration of temporal changes in metabolite concentrations and transport mechanisms within plants. The spatial information derived from these analyses is crucial for understanding the complex interactions that occur within plant tissues, offering significant contributions to various aspects of plant research. Recent studies have highlighted the utility of this approach in different crops, such as biofortified tomatoes, where spatiotemporal metabolomics has revealed the biosynthetic pathway of vitamin D, providing new insights into approaches for achieving vitamin D sufficiency [82]. Similarly, in strawberries, matrix-assisted laser desorption/ionization time-of-flight imaging mass spectrometry (MALDI-TOF IMS) has been used to visualize the distribution of metabolites at different maturity stages, revealing how compounds like citric acid and anthocyanins evolve during ripening [25]. In barley roots, comparative spatial lipidomics under salt stress has uncovered cellular lipid remodeling in response to environmental conditions [83], while in maize seeds, spatiotemporal metabolomics has provided detailed insights into the distribution of phospholipids and triacylglycerols during germination, highlighting developmental differences within embryos [84]. Moreover, spatial metabolomics has revealed the distribution patterns of small molecules, such as lipids and TCA cycle metabolites, in developing maize roots, providing valuable information on root growth and development [85].

Among these examples, studies focusing on lipids underscore the specific role of lipidomics within the broader context of spatial metabolomics. The integration of spatiotemporal metabolomics and lipidomics, particularly in analyzing plant responses to environmental stress or different developmental stages, offers a more holistic understanding of the spatial dynamics of biomolecules. This combined approach not only enhances our knowledge of plant physiology but also has significant implications for crop improvement strategies. Furthermore, integrating metabolomics with other omics approaches offers a comprehensive understanding of plant biology, allowing for the deeper exploration of complex interactions between metabolites, ions, and other biomolecules within plant systems. By leveraging the strengths of each omics technique, researchers can uncover new insights into plant growth, development, and responses to environmental factors, ultimately contributing to advancements in agriculture and nutrition.

## 4. Harnessing Metabolomics for Precision Crop Improvement

### 4.1. Multi-Omics Integration Empowers Precise Crop Improvement

**Figure 2:**
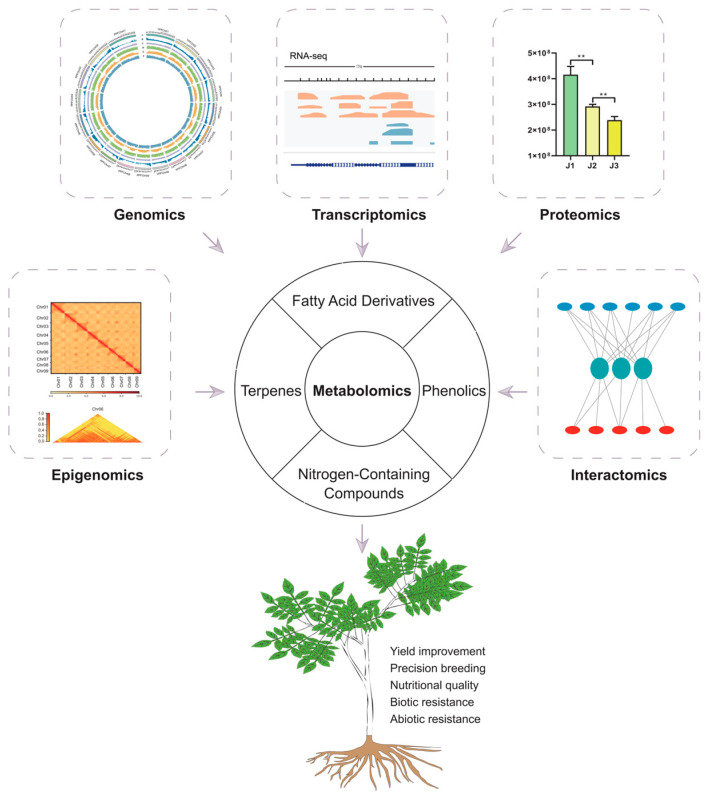
Integration of metabolomics with multi-omics approaches to enhance crop improvement. Note: ** means extremely significant difference (*p* < 0.01).

The integration of metabolomics with other omics approaches has significantly advanced crop improvement by providing a comprehensive understanding of plant biology at multiple levels. Unlike traditional breeding methods, which rely on limited genetic markers, multi-omics methods provide insights into complex traits at the genetic, transcriptomic, proteomic, and metabolic levels. This enables researchers to accelerate breeding processes and uncovers the molecular mechanisms underlying key agronomic characteristics. A systematic multi-omics workflow integrates genomics, transcriptomics, metabolomics, epigenomics, and proteomics data to elucidate the genetic and biochemical bases of traits (Figure 2). This approach combines various datasets to perform DEGs and DAMs analysis, time-series studies, and enrichment analyses (GO/KEGG), offering a comprehensive understanding of complex traits. The integration of population genetic approaches, such as GWAS and QTL mapping, with multi-omics data, further refines the identification of key genetic factors that influence crop phenotypes.

**Figure 3:**
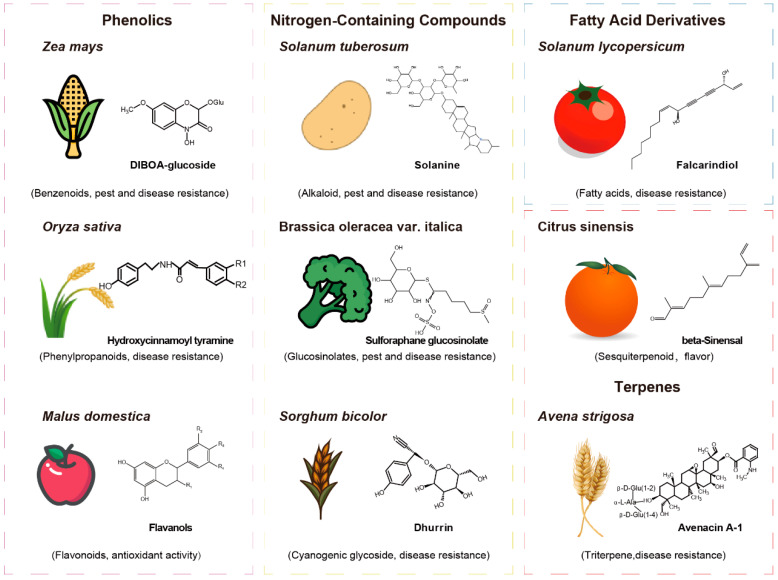
Diverse secondary metabolites across key crops with structural classes and biological roles.

While the multi-omics approach sheds light on the complex regulatory networks underlying plant traits, it is equally important to focus on specific metabolites that play a critical role in plant–environment interactions. These metabolites, particularly secondary metabolites, exhibit lineage specificity and are involved in crucial ecological functions, including defense mechanisms and responses to abiotic stress. Secondary metabolites, such as phenolics, nitrogen-containing compounds, fatty acid derivatives, and terpenes, are often lineage-specific and facilitate interactions with biotic and abiotic environments (Figure 3) [7,8,86]. These metabolites are essential for crop improvement, as they contribute to pest and disease resistance, nutritional quality, and flavor. Understanding their biosynthesis and function, along with their integration into multi-omics studies, is vital for developing enhanced crop varieties.

This comprehensive strategy enhances the effectiveness of crop breeding programs, aimed at developing new varieties with improved agronomic traits, such as resilience to both biotic and abiotic stresses, nutritional value, and overall productivity.

### 4.2. Secondary Metabolites in Defense Against Biotic Stresses

Secondary metabolites are the frontline chemical arsenal of plants against pests and pathogens. Their diversity and lineage-specificity underpin complex defense mechanisms. They include phenolics, nitrogen-containing compounds, and terpenes.

**Phenolics** are categorized into benzenoids, phenylpropanoids, and flavonoids, each serving distinct functions. In maize, benzenoids have been implicated in defense mechanisms against pathogens and insect resistance [87,88]. In rice, phenylpropanoids are associated with heightened resistance to the fungal pathogen *Magnaporthe oryzae* [89]. In apples, flavonoids are involved in the pigmentation of fruits and flowers, which is vital for attracting pollinators, while also providing protection against herbivores, bacterial and fungal infections, and mitigating environmental stressors like UV radiation [90].

**Nitrogen-containing compounds**, including alkaloids, glucosinolates, and cyanogenic glycosides, constitute a distinct and important category of secondary metabolites. Steroidal alkaloids (SAs) and their glycosylated forms, steroidal glycoalkaloids (SGAs), are predominantly found in the Solanaceae and Liliaceae families. These metabolites are particularly abundant in Solanaceae crops like potatoes, tomatoes, and eggplants. While SGAs are crucial for plant defense, providing resistance against various pathogens, including bacteria, fungi, and insects, they are also recognized for their anti-nutritional properties in humans due to their toxic effects [91,92]. Glucosinolates, primarily located in the Brassicaceae family, serve as key defensive compounds against herbivores and pathogens [93,94]. Cyanogenic glycosides are another example of nitrogen-containing secondary metabolites with a broad distribution among important food crops. This class of compounds is particularly effective as a chemical defense mechanism against herbivores, as exemplified by the cyanogenic glycosides found in Sorghum [95,96].

**Terpenes**, known for their aromatic properties, play a crucial role in plants by attracting pollinators and acting as defensive compounds against various pests and pathogens [97,98]. Exploring the roles of secondary metabolites through multi-omics analyses uncovers their contributions to plant fitness and stress responses. This approach enhances our understanding of key traits, such as pest resistance and nutritional quality, and informs crop improvement strategies. Collectively, the multi-omics dissection of these major defensive metabolite classes not only deepens our understanding of plant–herbivore/pathogen interactions but also pinpoints genetic and metabolic targets for engineering or breeding crops with enhanced, durable resistance to biotic stresses.

### 4.3. Metabolomic Reprogramming Under Abiotic Stresses

Plants constantly face diverse abiotic stresses that threaten their growth and productivity. To survive, they activate complex metabolic reprogramming, adjusting both primary and specialized metabolism to maintain cellular homeostasis. Multi-omics approaches have been instrumental in decoding these adaptive responses, revealing conserved and stress-specific metabolic signatures across crops.

**Drought and osmotic stress** primarily trigger the accumulation of osmoprotectants and antioxidants. In maize, integrated genomics and metabolomics have linked tolerance to salt-induced osmotic stress with the accumulation of compatible solutes like proline and specific flavonoids such as apigenin derivatives [37]. These metabolites aid in osmotic adjustment and ROS scavenging. Similarly, in tomato, a multi-omics study revealed that drought resistance is associated with phenolamide accumulation, regulated by two gene clusters and the transcription factor SlMYB13 [38]. These discoveries provide direct metabolic targets for breeding drought-resilient varieties.

**Salinity stress** involves ionic toxicity, osmotic imbalance, and oxidative damage. Research has highlighted the importance of lipid remodeling and microbial interactions. In rice, mGWAS of seed glycerolipids identified *OsLP1* as a key gene controlling saturated triacylglycerol levels; its allele is co-selected with a flooding tolerance QTL, linking lipid metabolism to submergence-associated stress tolerance [35]. Furthermore, wild soybean under salt stress enriches beneficial *Pseudomonas* in the root microbiome by exuding purine metabolites like xanthine, which acts as a chemoattractant to enhance microbial colonization and plant salt tolerance [68]. Ionomics-integrated research in rice exposed to platinum nanoparticles showed disruption of iron homeostasis and induction of oxidative stress, reflected in changes in organic acid and antioxidant metabolism [78].

**Temperature stress** affects membrane stability, protein function, and ROS generation. Multi-omics studies in potato, rapeseed, and peanut consistently show that cold tolerance is associated with the accumulation of protective compounds such as phenolics, flavonoids, and sugars (e.g., raffinose) [61]. In potato, salicylic acid priming enhances freezing tolerance by upregulating genes in phenylpropanoid and raffinose biosynthesis, leading to the accumulation of chlorogenic acid and raffinose [61]. Although heat stress is less covered in our cited studies, research on apple adaptation to altitude provides insights into combined stress (low temperature and high light): fruits at high altitude accumulated anthocyanins and other phenolics, along with changes in carbohydrate metabolism and photosynthetic protein abundance, indicating coordinated proteomic and metabolic adaptation [62].

Heavy metal and chemical stresses activate detoxification and sequestration pathways. In wheat seedlings, florfenicol stress caused the downregulation of photosynthetic proteins and the upregulation of TCA cycle and oxidative stress response proteins, accompanied by the accumulation of TCA cycle intermediates and GABA, indicating a shift from photosynthesis to respiration and nitrogen remobilization [61]. In apple under cadmium stress, dopamine application recruited beneficial microbes by altering root exudates, enhancing antioxidant activity and photosynthesis to alleviate toxicity [73]. Furthermore, in cassava, ionomics and microbiome analysis revealed that Lactococcus lactis improves potassium uptake and reduces aluminum toxicity by modulating the root ionome and metabolome [79].

**Nutrient stress** forces plants to optimize resource allocation and engage in symbiotic relationships. A study on lotus japonicus showed that under low nitrogen, bacterial Nod factors reprogram root exudate composition, which in turn shapes the rhizosphere microbiome to facilitate nitrogen acquisition [72]. Moreover, a multi-omics analysis of an agroecosystem demonstrated the significant contribution of organic nitrogen to crop yield, linking soil nutrient status with plant metabolic profiles [80].

In summary, the integration of metabolomics with multi-omics approaches has revolutionized our understanding of plant metabolic responses to environmental challenges. As illustrated in this section, plants employ a diverse arsenal of specialized metabolites for defense against biotic stresses. Concurrently, they orchestrate complex reprogramming of both primary and specialized metabolisms to cope with abiotic stresses such as drought, salinity, and extreme temperatures. By linking genetic variations to changes in gene expression, protein function, and metabolite production across these diverse conditions, multi-omics integration enables the precise identification of molecular targets for crop improvement. This comprehensive strategy is essential for developing next-generation crop varieties with enhanced, multi-faceted resilience to support sustainable agriculture in a changing climate.

## 5. Challenges and Future Perspectives in Metabolomics and Multi-Omics Integration

Metabolomics research faces several significant challenges, primarily due to the inherent complexity and diversity of metabolites in biological systems. The vast range of molecular entities, each with unique chemical properties and concentrations that can vary by several orders of magnitude, complicates comprehensive measurement and analysis. For instance, while metabolites such as phytohormones may be present at femtomolar concentrations, central metabolites exist in millimolar ranges [6]. Additionally, the subcellular compartmentalization of metabolites adds further complexity, making accurate detection and quantification even more challenging [14]. Although much of metabolomics research has focused on enhancing the coverage of the plant metabolome, the in vivo functions of the majority of plant metabolites remain poorly understood. Targeted metabolomics, which focuses on specific pathways, also faces substantial obstacles. No single analytical technique can cover the full spectrum of metabolites, and methods such as MS and NMR spectroscopy each have limitations. MS-based metabolomics, for instance, can suffer from issues like ion suppression and matrix effects, while NMR requires pure samples, making the analysis of minor metabolites in complex mixtures particularly challenging [99]. Additionally, data analysis remains a critical bottleneck, particularly in the identification of unknown metabolites and the validation of biomarkers. Inter-laboratory reproducibility is another major concern, as it is often difficult to replicate results across different settings due to the inherent variability in biological samples.

In addition to the challenges mentioned above, another key issue is the temporal delay in metabolomics relative to other omics approaches. Unlike transcriptomic or proteomic changes, which often occur more rapidly, shifts in the metabolome typically lag behind. This is because metabolites are the end products of complex biochemical pathways, and their synthesis, modification, and accumulation take time. As a result, metabolomic profiles tend to reflect downstream responses, rather than immediate changes in gene expression or protein activity, complicating the integration of metabolomics data with other omics in multi-omics studies. This temporal delay can pose difficulties in aligning and interpreting data across different omic layers, limiting the effectiveness of multi-omics approaches in capturing synchronized biological responses.

To address these challenges, future efforts should prioritize: (1) establishing standardized protocols and public metabolite databases to improve reproducibility; (2) developing computational tools that better integrate temporal and spatial multi-omics data; (3) applying genome editing to functionally validate omics-predicted gene–metabolite links; (4) harnessing microbiome–metabolome interactions for novel breeding strategies. These focused advances will broaden the applications of metabolomics in sustainable crop improvement.

## 6. Conclusions

Over the past two decades, metabolomics and multi-omics research have been extensively explored, significantly enhancing our understanding of plant metabolic diversity and its implications for crop improvement. This review emphasizes the practical applications of these approaches in improving crop traits, such as yield, nutritional quality, and stress resilience. Key conceptual advances emerging from this integration include: metabolic traits are governed by multi-layered regulatory networks spanning genetic, epigenetic, transcriptional, and post-translational levels; natural variation in metabolism often arises from coordinated changes in gene clusters and regulatory hubs; epigenetic modifications provide an environmentally responsive layer of regulation independent of DNA sequence; and microbiome–metabolome feedback loops extend the plant’s metabolic phenotype beyond its own genome. Building on these advances, promising future directions include mEWAS to capture environment-responsive trait variation, spatiotemporal metabolomics integrated with single-cell transcriptomics, machine learning for predicting metabolic outcomes from multi-omics inputs, and synthetic ecology to engineer microbiome–metabolome interactions for stress tolerance. However, critical challenges remain in annotating unknown metabolites, developing causal inference tools, and translating omics-discovered gene–metabolite links into validated breeding targets through genome editing. Addressing these challenges through continued innovation in analytical techniques and computational methods will be essential for realizing the full potential of multi-omics research in sustainable agriculture and global food security.

## Figures and Tables

**Table 1:** Metabolomics data analysis methods.

Purposes	Methods	References
Metabolome profiling, novel metabolite discovery (Metabolomics, spatiotemporal metabolomics)	Nuclear Magnetic Resonance (NMR)	[18]
	Mass Spectrometry (MS)	[20]
	Gas Chromatography–Mass Spectrometry (GC-MS)	[16]
	Liquid Chromatography–Mass Spectrometry (LC-MS)	[17]
	Capillary Electrophoresis–Mass Spectrometer (CE-MS)	[21]
	Isotope labeling mass spectrometry	[22]
	Direct Infusion Mass Spectrometry (DIMS)	[19]
	Liquid Chromatography–Ultraviolet Detector (LC-UV)	[23]
	Fourier Transform Ion Cyclotron Resonance Mass Spectrometry (FTICR-MS)	[24]
	Matrix Assisted Laser Desorption Ionization–Mass Spectrometry Imaging (MALDI-MSI)	[25]
	Ion Mobility Spectrometry (IMS)	[26]
	Ultra-High-Performance Liquid Chromatography High-Resolution Mass Spectrometry (UHPLC-HRMS)	[27]

## Data Availability

No data was used for the research described in the article.
